# Liver Enzyme Abnormalities and Associated Risk Factors in HIV Patients on Efavirenz-Based HAART with or without Tuberculosis Co-Infection in Tanzania

**DOI:** 10.1371/journal.pone.0040180

**Published:** 2012-07-11

**Authors:** Sabina Mugusi, Eliford Ngaimisi, Mohamed Janabi, Omary Minzi, Muhammad Bakari, Klaus-Dieter Riedel, Juergen Burhenne, Lars Lindquist, Ferdinand Mugusi, Eric Sandstrom, Eleni Aklillu

**Affiliations:** 1 Infectious Disease Unit, Department of Clinical Science and Education, Karolinska Institutet, Södersjukhuset, Stockholm, Sweden; 2 Department of Internal Medicine, Muhimbili National Hospital, Dar es Salaam, Tanzania; 3 Division of Clinical Pharmacology, Department of Laboratory Medicine, Karolinska University Hospital-Huddinge, Karolinska Institute, Stockholm, Sweden; 4 Unit of Pharmacology and Therapeutics, School of Pharmacy, Muhimbili University of Health and Allied Sciences, Dar es Salaam, Tanzania; 5 Department of Internal Medicine, Muhimbili University of Health and Allied Sciences, Dar es Salaam, Tanzania; 6 Department of Clinical Pharmacology and Pharmacoepidemiology, University of Heidelberg, Heidelberg, Germany; 7 Division of Infectious Diseases, Department of Medicine, Karolinska Institute at Karolinska University Hospital Huddinge, Stockholm, Sweden; Johns Hopkins University School of Medicine, United States of America

## Abstract

**Objectives:**

To investigate the timing, incidence, clinical presentation, pharmacokinetics and pharmacogenetic predictors for antiretroviral and anti-tuberculosis drug induced liver injury (DILI) in HIV patients with or without TB co-infection.

**Methods and Findings:**

A total of 473 treatment naïve HIV patients (253 HIV only and 220 with HIV-TB co-infection) were enrolled prospectively. Plasma efavirenz concentration and CYP2B6*6, CYP3A5*3, *6 and *7, ABCB1 3435C/T and SLCO1B1 genotypes were determined. Demographic, clinical and laboratory data were collected at baseline and up to 48 weeks of antiretroviral therapy. DILI case definition was according to Council for International Organizations of Medical Sciences (CIOMS). Incidence of DILI and identification of predictors was evaluated using Cox Proportional Hazards Model. The overall incidence of DILI was 7.8% (8.3 per 1000 person-week), being non-significantly higher among patients receiving concomitant anti-TB and HAART (10.0%, 10.7per 1000 person-week) than those receiving HAART alone (5.9%, 6.3 per 1000 person-week). Frequency of CYP2B6*6 allele (p = 0.03) and CYP2B6*6/*6 genotype (p = 0.06) was significantly higher in patients with DILI than those without. Multivariate cox regression model indicated that CYP2B6*6/*6 genotype and anti-HCV IgG antibody positive as significant predictors of DILI. Median time to DILI was 2 weeks after HAART initiation and no DILI onset was observed after 12 weeks. No severe DILI was seen and the gain in CD4 was similar in patients with or without DILI.

**Conclusions:**

Antiretroviral and anti-tuberculosis DILI does occur in our setting, presenting early following HAART initiation. DILI seen is mild, transient and may not require treatment interruption. There is good tolerance to HAART and anti-TB with similar immunological outcomes. Genetic make-up mainly *CYP2B6* genotype influences the development of efavirenz based HAART liver injury in Tanzanians.

## Introduction

Tuberculosis (TB) is the most common opportunistic infection and leading cause of morbidity and mortality in persons with HIV/AIDS in sub-Saharan Africa and worldwide. Overlapping toxicities, in particular drug-induced liver Injury (DILI) can complicate multidrug therapy of any kind. Concomitant anti-TB therapy significantly increases the risk of DILI [1], [2]. DILI may range from transient asymptomatic elevation of liver enzymes to fulminant liver failure requiring treatment interruption, and the subsequent adherence problem may cause treatment failure, relapse or drug resistance [3]–[5]. Efavirenz based HAART is the first drug of choice to be given with rifampicin based anti-TB therapy in HIV-TB co-infected patients [6]. Though effective, there is growing concern about efavirenz-based HAART associated liver injury. Cases of acute liver failure associated with efavirenz-based HAART requiring liver transplantation are reported [7], [8]. Higher risk of severe DILI among Hispanic HIV-infected patients after initiation of HAART which is mainly due to NNRTIs has been reported recently [9].

Efavirenz, the recommended NNRTI for co-treatment with rifampicin in resource-limited settings is metabolized in the liver mainly by CYP2B6 enzyme and to a lesser extent by CYP3A4/5 [10]. Rifampicin, a potent inducer of these enzymes, reduces plasma efavirenz concentrations. P-glycoprotein and OATP1B1 coded by *ABCB1*and *SLCO1B1* gene respectively play a key role in the transportation of anti-TB drugs including rifampicin. The genes coding for these drug metabolizing enzymes and transporters are inducible by rifampicin and are polymorphic displaying wide inter individual and inter-ethnic variation in enzyme or transporter activity. *CYP2B6 516G>T* (CYP2B6*6), a defective variant allele associated with high efavirenz plasma concentration, CNS side effects and altered enzyme inducibility occurs at a higher frequency (up to 40%) in sub-Saharan African populations; most being either heterozygous or homozygous genotype [11]–[15]. *CYP3A5* is polymorphically expressed in black populations (60%) with common and specific defective variant alleles [16]. Significant influence of *CYP3A5* variants alleles (CYP3A5*3, *6 and *7) on quinine metabolism in Tanzanians is reported [17]. Association of genetic polymorphism in *ABCB1, CYP3A5* and *SLCO1B1* with variation in susceptibility to adverse drug reaction and toxicity are reported previously [18]–[20]. Recently we reported significant differences in *SLCO1B1* variant alleles (SLCO1B1*1B, *5 and *15) between Tanzanians and Europeans [21].

Recent studies provide evidence for implication of pharmacogenetic variation in determining susceptibility to DILI [22], [23]. Incidence and predictors of DILI varies between populations partly due to genetic or geographical variations [22]–[24]. African populations are genetically too heterogeneous [25] making it impossible to extrapolate genetic information from one population to the other and hence there is a need for more studies to identify the aetiologies and risk factors for better management of antiretroviral and anti-tuberculosis DILI and to design prevention strategies. In this study we describe the incidence of liver enzyme abnormalities and the associated risk factors including socio-demographic and baseline biochemical characteristics, pharmacokinetic and pharmacogenetic predictors in Tanzanian HIV patients receiving efavirenz based HAART with or without concomitant rifampicin based anti-TB therapy.

## Methods

### Ethics Statement

The study was ethically approved by the Institutional Review Board (IRB) of the Muhimbili University of Health and Allied Sciences (MUHAS) in Dar es Salaam, Tanzania and by the Karolinska Institutet in Stockholm, Sweden. Prior written informed consent was obtained from all study participants.

### Study Population and Setting

This is part of a larger prospective cohort study funded by European and Developing Countries Clinical Trial Partnership (EDCTP) entitled “Optimization of HIV and TB co-treatment based on pharmacokinetic and pharmacogenetic aspects of drug- drug interactions between rifampicin and efavirenz”. The study was conducted between September 2007 and June 2010 at Muhimbili National Hospital (MNH), Infectious Disease Centre (IDC) and Mwananyamala Municipal Hospital all within Dar es Salaam city, Tanzania. The study design, population, inclusion and exclusion criteria’s plus detailed description of this cohort study has been described previously [26]. In brief, the study design was descriptive, non-randomized, parallel assignment, prospective cohort to evaluate the incidence and predictors of DILI in HAART naïve HIV patients. A total of 473 patients were enrolled in parallel and assigned into two different types of treatment groups depending upon the disease conditions and type of treatment to receive according to the national TB and HIV treatment guideline during the study period namely; 255 HIV patients without TB co-infection (referred to as HIV only) plus 221 HIV patients co-infected with TB (referred to as HIV-TB). The eligibility criteria were age ≥18 years, CD4 count <200 cells/µL and not on other known hepatotoxic drugs concurrently (except co-trimoxazole, 960 mg per day, which was given for all participants before enrolment and during the follow up period according to the treatment guideline). None of the participants received treatment for tuberculosis five years before enrolment. Exclusion criteria were pregnant women, prisoners and a low Hb (≤8 g/dL). After receiving informed consent, complete history and physical examination were taken at baseline and at scheduled visits. Patients were followed up for a period of 48 weeks for HIV only; and 52 weeks for HIV-TB at regular intervals. All patients received the usual care for HIV and TB, and any other opportunistic infections in the Care and Treatment Centers (CTC) as indicated in the guidelines.

HIV only infected patients were initiated on an efavirenz based HAART regimen with two nucleoside reverse transcriptase inhibitors (NRTI’s). In HIV patients co-infected with TB, a rifampicin based anti-TB therapy was initiated 4 weeks prior to the initiation of efavirenz based HAART and 2 NRTI’s. Clinical and laboratory follow up were conducted at predetermined intervals (0, 1, 2, 4, 6, 8, 12, 24, 36 and 48 weeks). According to the CIOMS (Council for International Organizations of Medical Science) definition, DILI generally occurs within 5–90 days after drug ingestion [27]. Likewise prospective studies from Africa reported the median and range of HAART and/or anti-TB DILI onset to be within 90 days after initiation of therapy [28]–[30]. Hence for the purpose of this study censoring was done at 12 weeks.

### Data Collection and Laboratory Analysis

Socio-demographic characteristics, detailed history of present and past illnesses were recorded with findings from a general physical examination using a case record format and questioner prepared for the study. Clinical data was collected at baseline, and at other pre-determined intervals of 1^st^,2^nd^,4^th^,8^th^,12^th^, 24^th^, 36^th^ and 48th week (for HIV only patients) and 52 weeks (for TB-HIV confected patients) after therapy initiation. The study physicians did clinical evaluations for any adverse events and patients’ progress at every clinical visit. The laboratory investigations were done according to the same clinical schedule in both treatment groups. A verbal autopsy questionnaire was administered by the clinician to the relatives of the deceased for reported deaths during the study period to ascertain cause of death [31], [32] Where available, information from the deceased’s death certificate was used to complete the verbal autopsy questionnaire.

The routine laboratory testing was performed at the Central Pathology Laboratory in MNH, including complete blood count, CD4 cell count, viral load assessment, Aspartate Aminotransferase (AST), Alanine aminotransferase (ALT), Alkaline phosphatase (AlkP), and total and direct bilirubin levels. In addition hepatitis B surface antigen, hepatitis C serology and VDRL were also done. Determination of CD4 cell count and HIV viral load was done before starting HAART and at week 12, 24 and 48 on therapy. Laboratory tests for liver enzymes were performed before starting therapy and on the 1^st^, 2^nd^, 4^th^, 8^th^, 12^th^, 24^th^ and 48^th^ weeks after initiation of treatment. The serum biochemistry for liver enzymes was determined using a COBAS MIRA chemistry analyzer (GMI, MI, USA) after it was calibrated. The determination of hepatitis B surface antigen (HBsAg) and anti-hepatitis C virus IgG antibody (anti HCV) was done using the antibody capture ELISA (Adaltis – EIAgen kit).

### CYP2B6, CYP3A5, ABCB1 and SLCO1B1 Genotyping

Genomic DNA was isolated from peripheral blood leukocytes using QIAamp DNA Maxi Kit (QIAGEN GmbH. Hilden. Germany). Genotyping was carried out at the division of clinical pharmacology, Department of laboratory medicine, Karolinska University Hospital-Huddinge, Karolinska Institute Stockholm, Sweden. Genotyping for SNPs were done by real time PCR using pre-developed Taqman assay reagents for allelic discrimination (Applied Biosystems Genotyping Assays) according to the manufacturer’s instructions. Allelic discrimination reactions were performed using TaqMan**®** (Applied Biosystems, CA, USA) genotyping assays with the following ID number for each SNP: (C__7586657_20 for *ABCB1* 3435C>T rs1045642, C__11711730_20 for *CYP2B6* c.516G>T rs3745274 [CYP2B6*6], C__26201809_30 for *CYP3A5* 6986A>G rs776746 [CYP3A5*3], C__30203950_10 for *CYP3A5* g.14690G>A rs10264272 [CYP3A5*6], C__32287188_10 for *CYP3A5* g.27131_27132insT rs41303343 [CYP3A5*7] on ABI 7500 FAST (Applied Biosystems, Foster City, CA). The final volume for each reaction was 10µl, consisting of 2x TaqMan Universal PCR Master Mix (Applied Biosystems), 20 X drug metabolising genotype assay mix and 10 ng genomic DNA. The PCR profile consisted of an initial step at 50°C for 2 min and 50 cycles with 95°C for 10 minutes and 92°C for 15 sec. Genotyping for SLCO1B1 388A>G (rs2306283) and 521T>C (rs4149056) was done using LightCycler® based method as described previously [21]. Haplotype analysis was done using Haploview v.4.1 software.

### Quantification of Plasma Efavirenz Concentration

On the 4^th^ week of efavirenz-based HAART, 8 ml of blood were collected 16 hrs post efavirenz dosing, centrifuged, and 2 mL plasma aliquot was taken and stored at −80°C for determination of efavirenz and its metabolite concentration. Plasma samples were sent in dry ice to the Department of Clinical Pharmacology and Pharmacoepidemiology, University of Heidelberg, Germany. The determination of plasma efavirenz and 8-hydroxyefavirenz concentrations by LC/MS/MS was performed as described previously [13], [14]. The lower limits of quantification in plasma were 10.0 ng/mL for efavirenz and 0.4 ng/mL for 8-hydroxyefavirenz.

#### Case definition

Identification of DILI was according to the CIOMS criteria, which is based on selected laboratory liver parameters (CIOMS laboratory criteria) and the exclusion of any disease-related causes of liver injury [27]. Patients with DILI were defined as having ≥2 times the upper normal limit (UNL) of AST and/or ALT. We used 50 U/L as an UNL for AST and ALT while 1.5 mg/dL was for bilirubin. Severe DILI was defined as AST and/or ALT level >5 times the UNL. This was based on the WHO grading system for monitoring of laboratory toxicities adopted in the National HIV treatment guidelines; Grade I (Mild) 1.23–2.5x UNL, Grade II (Moderate) >2.5–5.0x UNL, Grade III (Severe) >5.0–10.0x UNL and Grade IV (Potentially life threatening) >10.0X UNL).

### Statistical Methods

The clinical assessment and laboratory results that were recorded into a Microsoft Access database were analyzed using Statistical Package for the Social Sciences (PASW – former SPSS) version 18 and R version 2.9.2 (R Foundation for Statistical Computing, Vienna, Austria). *P* value of <0.05 was considered statistically significant. Descriptive statistics for the baseline demographic and clinical characteristics and the laboratory values at baseline and through to the first 12 weeks were tested with the independent t-test and χ2-test. Multiple imputation (MI), using predictive mean matching, was performed n = 10 times in order to avoid bias due to baseline characteristics missing at random (MAR). All statistical calculations, except the descriptive statistics, were performed on the imputed data with imputation-corrections to the resulting standard errors.

Univariate and multivariate Cox proportional hazards regressions, using the Efron method for tie handling, were performed. The variables included in the multivariate model were those with either a theoretical importance or ones with a p-value<0.05 in the univariable models. Interactions with group were tested for within the multivariable model. Normality of kinetic data was assured by transforming the data to Log 10 values before statistical analysis. Interactions with HIV/TB co-infection or HIV only were tested for within the multivariate model. The efavirenz metabolic ratio (EFV MR) was calculated by dividing concentrations of efavirenz by 8-hydroxyefavirenz. The verbal autopsy was used to determine the probable cause of death, by asking the relatives about the events leading up to the death of the patient.

## Results

A total of 486 newly diagnosed HAART naïve patients were recruited prospectively and followed up to 48 weeks. For the purpose of this study 13 patients were excluded due to missing laboratory results. Baseline demographic and clinical data, plus laboratory results of 473 patients at 12 weeks were used for analysis. An additional twenty six (5.3%) patients with elevated baseline ALT and or AST levels at baseline were not evaluated because their elevated liver enzymes could be due to other factors apart from HAART or anti-TB drugs.

### Onset of DILI

There were consistently higher AST and ALT levels at weeks 1,2,4,6,8 and 12 in the patients with than without DILI. In the HIV only patients the highest peak of AST or ALT levels was observed at one week. But in the TB-HIV group, two peak time points for DILI was observed; one week after starting anti-TB therapy followed by another peak corresponding to one week after starting HAART. Over all the median time to DILI in HIV only patients was 2 weeks while in the TB-HIV co-infected patients the median time to DILI was 5 weeks corresponding to 1 week after HAART was added on to the anti-TB therapy. This difference was not statistically significant (p = 0.07). None of the patients had a DILI onset after week 12.

### Incidence of DILI and Effect of Treatment Type

**Figure 1:**
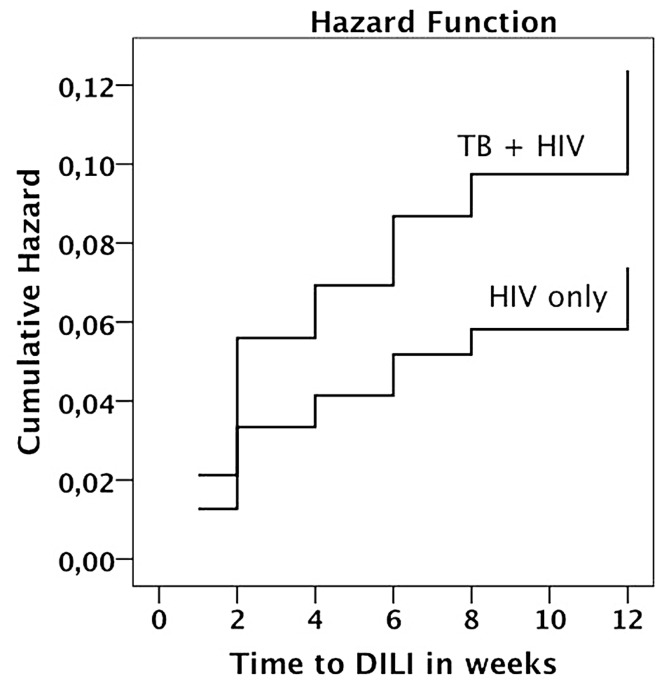
Kaplan-Meier curves indicating estimate cumulative hazard for the development of drug induced liver injury between HIV patients receiving efavirenz based HAART alone and TB-HIV coinfected patients receiving efavirenz based HAART with rifampicin based anti-TB therapy during the first three months of follow up period.

Data from 253 HIV only patients and 220 HIV/TB co-infected patients was used for analysis. Thirty-seven (7.8%) patients were defined as having DILI based on one or more AST/ALT levels ≥2 X UNL in the whole study population. Kaplan-Meier plot indicating cumulative hazard for the development of DILI stratified by treatment group is presented in Figure 1. Fifteen (5.9%, 6.3 per 1000 person-week) patients developed DILI among those receiving efavirenz based HAART alone (HIV only) while twenty-two (10.0%, 10.7 per 1000 person-week) patients receiving concomitant efavirenz based HAART and rifampicin based anti-TB therapy (HIV-TB group) developed DILI. Though not significant (p = 0.07), the incidence of DILI in patients on concomitant HIV and TB therapy was almost two folds compared to those treated with efavirenz based HAART only. Comparison of socio-demographic and baseline biochemical characteristics between patients with and without DILI are presented in Table 1.

**Table 1:** Description of demographic and baseline laboratory characteristics of patients with and without DILI.

Characteristic			No DILI (n = 436)		DILI (n = 37)	Missing
Group	HIV only		238 (54.6%)		15 (40.5%)	0
	TB-HIV		198 (45.4%)		22 (59.5%)	
Sex	Females		254 (58.2%)		16 (43.2%)	0
	Males		182 (41.8%)		21 (56.8%)	
Age (Mean±SD)			39.74±9.19		41.81±10.98	5
BMI (Mean±SD)			20.97±4.21		20.56±3.58	39
Marital status	Single, divorced or widowed		237 (54.4%)		21 (56.8%)	0
	Married or Cohabiting		199 (45.6%)		16 (43.2%)	
Education status	Illiterate, able to read or write or primary		334 (76.6%)		31 (83.8%)	0
	Secondary or tertiary		102 (23.4%)		6 (16.2%)	
Karnofsky scores	90–100%		332 (76.1%)		23 (67.7%)	0
	≤80%		104 (23.9%)		11 (32.3%)	
WHO clinical stages	Stage II		193 (44.3%)		11 (29.7%)	0
	Stage III		218 (50.0%)		26 (70.3%)	
	Stage IV		25 (5.7%)		0	
HAART initiated	D4T+3TC+EFV		138 (32.0%)		14 (40.0%)	7
	AZT+3TC+EFV		293 (68.0%)		21 (60.0%)	
***Laboratory Parameters***						
Hepatitis B		Negative	307 (89.5%)	27 (90.0%)		100
		Positive	36 (10.5%)	3 (10.0%)		
Hepatitis C		Negative	347 (97.5%)	28 (90.3%)		86
		Positive	9 (2.5%)	3 (9.7%)		
VDRL		Negative	316 (93.2%)	33 (97.0%)		98
		Positive	23 (6.8%)	1 (3.0%)		
CD4 Cell counts (Mean±SD)		Baseline	100.26±66.58	78.59±58.87		0
		12 weeks	212.73±128.64	171.25±145.64		157
Mean Viral Load Log ± SD		Baseline	5.67±5.96	5.45±5.5		146
		12 weeks	5.22±5.74	5.0±5.3		425
Log efavirenz conc 4 wks after HAART (Mean ± SD)			3.4±3.36	3.33±3.22		166

### Clinical Presentation of DILI

There were proportionately more males who had DILI than women (10.3% and 6% respectively) though this was not statistically significant. There was a significant difference in the baseline WHO clinical stage with most of the patients being in WHO clinical stage III while none of the patients with DILI were in WHO stage IV (p = 0.039). There was a significant difference in clinical history of weight loss among those patients who developed DILI (p = 0.016). Alcohol consumption and use of Fluconazole tablets for treatment of oro-oesophageal candidiasis were not associated with the development of DILI. Eight patients (36.4%) of the HIV-TB group and 3 patients (20%) of the HIV only developed WHO grade III DILI. The rest of the patients were WHO grade II, and there was no patient with WHO grade IV DILI. Monitoring was done for both clinical and laboratory parameters and no treatment interruption were required in these patients.

The prevalence of HBsAg and anti HCV antibodies was 10.4% and 3.1% respectively. Twenty five percent (3 out of 12) of the patients with positive HCV antibody developed DILI (p = 0.028). Patients who developed DILI had lower baseline CD4 cell counts compared to those without DILI (p = 0.056).

### Effect of Pharmacogenetic Variations on DILI

**Figure 2:**
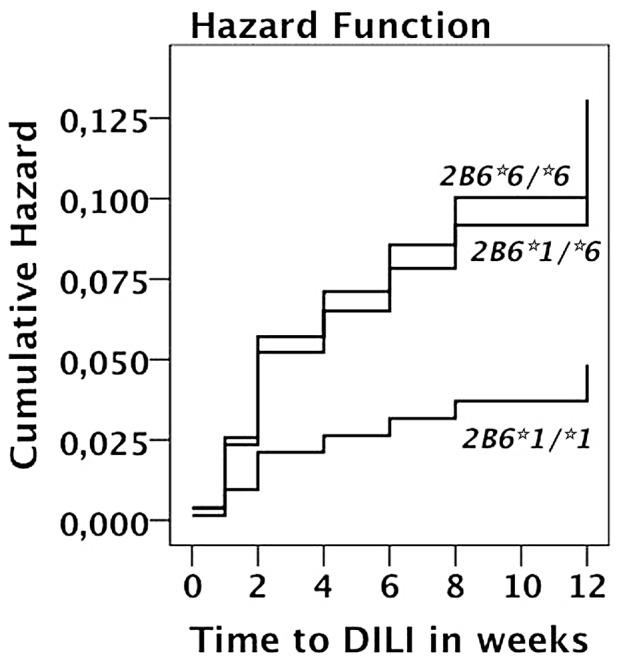
Kaplan-Meier curves indicating estimate cumulative hazard for the development of drug induced liver injury between the different CYP2B6*6 genotypes in patients receiving efavirenz based HAART with or without rifampicin based anti-TB therapy during the first three months of follow up period.

The association of pharmacogenetic variations in *CYP2B6*, *ABCB1*, *CYP3A5* and *SLCO1B1* genes with the development of DILI is presented in Table 2. Genotype data was obtained from 351 patients (209 HIV only, and 142 HIV-TB patients). The frequency of the defective variant allele CYP2B6*6 was significantly higher among patients with DILI (p = 0.03). The distribution of *CYP2B6* genotype was different between patients with and without DILI (p = 0.069). The proportion of subjects with CYP2B6*1/*1 genotype was significantly lower in patients with DILI (21%) than without DILI (44%), while those with CYP2B6*6/*6 were higher in DILI patients (21%) than patients without DILI (15%). Kaplan-Meier plot indicating cumulative hazard for the development of DILI stratified by CYP2B6*6 genotype is presented in Figure 2. For ABCB1 3435C/T there were no patients that developed DILI with TT genotype, while there were proportionately higher CC (8.3%) compared to CT (7.8%) among those with DILI.

**Table 2:** Comparisons of *CYP2B6*, *CYP3A5*, *NAT2*, *SLCO1B1* and ABCB1 variant alleles and genotype/haplotypes between DILI non-DILI cases among HIV patients with or without tuberculosis.

Gene	Allele/genotype/haplotype		No DILI	DILI	p-value
*CYP2B6*	allele	*1	414 (64.5%)	28 (50%)	0.031
		*6	228 (35.5%)	28 (50%)	
	genotype	*1/*1	141 (43.9%)	6 (21.4%)	0.069
		*1/*6	132 (41.1%)	16 (57.1%)	
		*6/*6	48 (14.9%)	6 (21.4%)	
*ABCB1 3435C/T*	allele	*T*	97 (15.1%)	7 (12.5%)	0.59
		*C*	545 (84.9%)	49 (87.5%)	
	genotype	*TT*	7 (2.9%)	0	0.72
		*CT*	83 (25.8%)	7 (25%)	
		*CC*	231 (72%)	21 (75%)	
*CYP3A5*	allele	*1	332 (51.1%)	24 (42.9%)	0.23
		None *1	318 (48.9%)	32 (57.1%)	
	genotype (number of *1 allele)	Zero	87 (26.8%)	5 (17.9%)	0.50
		One	158 (48.6%)	14 (50%)	
		Two	80 (24.69%)	9 (32.1%)	
*SLCO1B1*	allele	*1a	86 (13.6%)	5 (9,3%)	0.56
		*1b	525 (83.1%)	48 (88.9%)	
		*15	21 (3.3%)	1 (1.9%)	
	genotype	*1a/*15	5 (1.6%)	0	0.84
		*1a/*1a	7 (2.2%)	0	
		*1a/*1b	67 (21.2%)	5 (18.5%)	
		*1b/*15	16 (5.1%)	1 (3.7%)	
		*1b/*1b	221 (69.9%)	21 (71.8%)	

Efavirenz concentration data at 4 weeks after HAART initiation was obtained from 292 patients (174 HIV only, 118 HIV-TB group). The HIV only patients had higher plasma efavirenz concentrations (median = 1875 ng/mL; IQR: 1235–4024 ng/mL) compared to the HIV-TB group (median = 1481 ng/mL, IQR: 959–2327 ng/mL), this difference was statistically significant (p = 0.04). Similarly the body mass index (BMI) was different between HIV only patients and those with HIV-TB (p<0.0001). The use of HAART alone or anti-TB plus HAART and BMI were considered to investigate correlation between efavirenz plasma level and incidence of DILI. Full factorial ANOVA displayed a nearly significant interaction between development of DILI, BMI group and patients with HIV only or HIV-TB (p = 0.063, observed power = 46%, alpha = 0.05) with respect to plasma efavirenz concentration. The highest mean plasma efavirenz level was observed in DILI cases with BMI <18.5 who received efavirenz based HAART alone.

### Predictors of DILI

Univariate analysis was done using Cox Proportional hazard regression analysis for all variables in Table 3. History of weight loss, presence of nausea and or vomiting, Karnofsky scores of <80%, baseline AST, hepatitis C antibody positive and low baseline CD4 cell counts were seen to be predictors. In the multivariate model, the predictors of developing DILI include anti HCV positive (Hazards Ratio (HR) = 5.32, 95% Confidence interval (CI) = 1.02–27.82) and CYP2B6 *6/*6 (HR = 2.82, 95% CI = 1.04–7.65). All variables included in the multivariate model were tested for interactions with group, but no interaction was statistically significant.

**Table 3:** Univariate and Multivariate Cox proportional regression analysis to show the risk factors for developing DILI.

Variable	Univariate analysis		Multivariate Analysis	
	HR (95% CI)	p-value	HR (95% CI)	p-value
HIV and TB co-infection	1.68 (0.87–3.24)	0.120	1.28 (0.61–2.68)	0.513
Male sex	1.81 (0.94–3.46)	0.074	1.55 (0.73–3.27)	0.25
Age	1.02 (0.99–1.05)	0.274	1.01 (0.97–1.04)	0.774
Weight loss (ref – yes weight loss)	2.33 (1.20–4.54)	0.012	1.72 (0.83–3.56)	0.142
Nausea/Vomiting (ref – yes nausea/Vomiting)	2.58 (1.13–5.88)	0.024	2.25 (0.86–5.90)	0.100
BMI	0.97 (0.89–1.05)	0.419		
Alcohol consumption (ref – Yes alcohol use)	0.90 (0.41–1.98)	0.802		
Use of Fluconazole tablets (ref – Yes)	1.011 (0.24–4.20)	0.988		
WHO clinical stage (ref – WHO stage III & IV)	1.84 (0.91–3.72)	0.090		
Karnofsky Scores (ref – Karnofsky score <80%)	2.25 (1.16–4.38)	0.017	1.92 (0.88–4.15)	0.099
Baseline AST	1.01 (1.00–1.02)	0.007	1.00 (0.98–1.02)	0.905
Baseline ALT	1.01 (1.00–1.03)	0.060	1.01 (0.99–1.03)	0.371
Baseline AlkP	1.00 (1.00–1.01)	0.314		
Baseline Bilirubin (Total)	1.01 (0.99–1.02)	0.247		
Baseline Bilirubin (Direct)	0.98 (0.89–1.09)	0.737		
Hepatitis B positive (ref – HbsAg positive)	0.85 (0.26–2.77)	0.782	1.04 (0.30–3.55)	0.953
Hepatitis C positive (ref – HCV Ab positive)	3.69 (1.17–11.68)	0.026	4.91 (1.24–19.49)	0.024
Baseline CD4 cell count	0.99 (0.99–1.00)	0.023	1.00 (0.99–1.00)	0.110
CYP2B6*6 allele (ref carriers)	1.95 (0.85–4.45)	0.115	2.54 (1.04–6.20)	0.040
*ABCB1* (ref –3435 C/C)	1.18 (0.48–2.92)	0.723		
*CYP3A5* (ref – *0/*0)		0.747		
*CYP3A5* *1/*0	1.36 (0.52–3.56)	0.534		
*CYP3A5* *1/*1	1.58 (0.48–5.18)	0.452		
*SLCO1B1* (ref – absence of *15)	1.58 (0.19–13.01)	0.669		
Log HIV RNA Viral load at baseline	0.97 (0.82–1.14)	0.705		
Log efavirenz concentration at 4^th^ week	0.93 (0.69–1.25)	0.629		

### Effect of DILI on Treatment Outcome

There was a gradual and comparable rise in CD4 cell counts and gain in body weight between patients with and without DILI from baseline to 12 weeks after HAART initiation. The mean increase in CD4 cell count in patients with DILI after 12 weeks was 92 cells/ µL vs 112 cells/µL in those without DILI. There was no difference in the mean increase in CD4 after 12 weeks between the HIV only patients (113cells/ µL) and the HIV-TB (109cells/µL).

By the end of the study period, 13% (7 out of 54) of the patients that had died were defined as DILI with the median time to death of these 7 patients being 4 weeks (Interquartile range (IQR) = 6). Among those without DILI 47 died at the end of the study period. However, from the verbal autopsy reports these patients died of other causes than DILI. Despite the high pill burden especially in those on both anti-TB and HAART, patients self reported adherence was high (>95%). The 26 patients with elevated AST/ALT levels at baseline had an average mean decrease of 69.82 U/L from baseline to 12 weeks compared to those with initially normal values. Three of these 26 patients died by the end of the study period, with none of the deaths being related to DILI.

## Discussion

In the present prospective study we investigated the incidence and possible predictors of DILI following efavirenz based HAART with or without rifampicin based anti-tuberculosis treatment. Our results indicate that HAART and anti-TB DILI does occur with an overall prevalence being 7.8% and is well tolerated among the patients. The low incidence and tolerability of DILI among Tanzanian HIV patients is comparable with those reported from other studies in Africa [33]–[36]. DILI, as seen by the highest peaks of AST and/or ALT, developed fairly early during the course of treatment (one week after HAART initiation) in patients using HAART alone or with anti-TB plus HAART, similar to that reported elsewhere [37]. The DILI seen is transient, with AST and/or ALT levels coming back to within normal limits by 12 weeks of HAART. Patients with grade III DILI according to WHO guidelines were few, with none of our patients presenting with Grade IV DILI. Monitoring of the clinical and laboratory parameters was done and no medical intervention or treatment interruption was required in these patients. Our results indicate that Hepatitis C infection and CYP2B6*6 genotype are risk factors for the development of DILI.

The reported incidence of HAART and/or anti-TB DILI varies between African populations. The available few studies in general indicate lower incidence and good tolerability. A recent study from Uganda reported low incidence of severe hepatotoxicity within three months of first-line HAART and concluded that routine measurement of transaminases may not be necessary in all patients initiating HAART in resource limited settings [33]. Unexpectedly low (2%) anti-tuberculosis drug-induced hepatotoxicity was also reported in HIV-infected pulmonary tuberculosis patients from Malawi [38]. Antituberculosis drug-induced hepatotoxicity is reported to be uncommon (1%) in Tanzanian hospitalized pulmonary TB patients [39]. In contrast higher incidence of rifampicin based anti-TB DILI (17%) [28], efavirenz based HAART associated DILI (15%) [29] and concomitant HAART and anti-TB DILI (30%) from Ethiopia is reported [30]. Thus the incidence and predictors of DILI vary within the African population and hence caution needs to be applied in direct extrapolation of findings from one study population to another in resource-limited countries.

We observed relatively higher incidence of DILI in patients receiving efavirenz based HAART together with rifampicin based anti-TB (10%) compared to those receiving HAART alone (6%) though this was not statistically significant. Our result is in agreement with the previous reports describing concomitant HAART and anti-TB therapy exacerbates the incidence of DILI from Africa or elsewhere [1], [2], [33], [35], [40], [41].

In a subgroup of HIV TB co-infected patients, pronounced DILI was observed before the introduction of HAART indicating that rifampicin may contribute to the hepatotoxicity, however in the majority of cases the DILI appeared later in conjunction with the introduction of efavirenz.

Efavirenz is mainly metabolized by CYP2B6 enzyme. In the present study we found significant differences in the distribution of *CYP2B6* genotype between patients with and without DILI. The frequency of CYP2B6*6 (c.516TT) and CYP2B6*6/*6 genotype was over represented among patients with DILI. Similar findings were reported in a study from Ethiopia [29], [30]. CYP2B6*6 is associated with higher plasma efavirenz concentration and occurs at a high frequency in native African populations [12], [13], [15], [42]. CYP2B6*6 and high plasma efavirenz concentration are associated with adverse events mainly Central Nervous System (CNS) toxicity in HIV patients [11]. Recently we reported effect of *CYP2B6* genotype and wide between patient variability in plasma efavirenz concentration among Tanzanian HIV patients receiving efavirenz based HAART with or without rifampicin based anti-TB therapy [12], [13]. We found CYP2B6*6, a variant allele associated with higher efavirenz plasma concentration, as a risk factor for DILI but not with plasma efavirenz concentration itself. The incidence of efavirenz based HAART induced DILI (6%) in Tanzanian HIV patients is much lower compared to the 15% incidence reported in Ethiopian HIV patients treated with the same regimen and assessed with similar DILI case definition [29], [30].

Interestingly plasma efavirenz concentration was much higher in patients with low BMI receiving efavirenz based HAART alone compared to those with BMI>18.5 at baseline. Such a difference was not observed in patients receiving concomitant rifampicin based anti-TB therapy. In the present study we found no significant association of DILI with *CYP3A5*, *ABCB1 3435C>T* and *SLCO1B1* genotype.

We investigated the prevalence of hepatitis B and C virus infection in patients infected with HIV and the risk of HAART and anti-TB DILI in HIV only and HIV-TB co-infected patients. Viral hepatitis is common in Tanzanian HIV patients. The prevalence of Hepatitis B antigen and Hepatitis C antibody was 10.4% and 3.1% respectively which is similar to that described by other authors in the same setting [43]. In patients with chronic hepatitis B or C infections, the elevations in liver aminotransferases after initiating HAART might be due to drug toxicity or immune reconstitution inflammatory syndrome (IRIS) manifested as paradoxical hepatitis flare but distinguishing one from the other remains a challenge [44]. However we did not observe effect of hepatitis B co-infection but instead hepatitis C coinfection was a significant risk factor for DILI. In HIV-HBV-coinfected patients, in addition to antiretroviral therapy both Lamivudine and Tenofovir promptly inhibit HBV replication and lower HBV DNA viral load as well as alanine aminotransferase [45], [46]. All our study subjects received Lamivudine as part of anti-HIV regimen and this might lead to alleviating HBV co-infection as a risk factor for DILI. Our study demonstrates pre-existing liver injury due to hepatitis C virus as a risk factor for developing HAART and anti-TB DILI in HIV patients. A similar finding in HIV-TB co-infected patients is reported recently [47]. Co-infection with hepatitis C or HIV increases the relative risk of developing anti-TB drug induced hepatotoxicity by five and four fold respectively [48]. Therefore regular monitoring of liver enzymes and close clinical follow up is recommended during HAART and anti-TB therapy in patents co-infected with HIV and hepatitis C virus.

Gradual increase in CD4 cell counts and weight gain from baseline to 12 weeks indicates that clinical and immunologic benefits were comparable among patients receiving efavirenz based HAART with or without rifampicin [49]. Unlike other studies that show an older age group, alcohol consumption and use of Fluconazole tablets as a risk factor for the development of DILI, in this study no such association was observed [50], [51].

Recently the combined use of therapeutic drug monitoring (TDM) with pharmacogenetic testing is advocated to optimize efavirenz therapy [52]. Regular TDM practice is not feasible in resource-limited countries like Tanzania. Hence genotyping practice in HIV clinics could be another option to identify individuals who are at risk of developing efavirenz associated CNS toxicity and liver injury.

In summary, efavirenz based HAART and rifampicin based DILI does occur in Tanzanian HIV patients, presenting early in treatment. In majority of the cases, liver enzyme elevations after initiation of therapy are mild, transient and may not require modification of treatment. HCV positive, WHO clinical stages, history of weight loss and CYP2B6*6 genotype are significant predictors of DILI. Median time to DILI was 1 week after HAART initiation. Despite presence of DILI, there is good tolerance to HAART and anti-TB regimen with good clinical and immunological outcomes. We therefore encourage clinicians to avoid reluctance of combining HAART and anti-TB therapy for fear of toxicity in Tanzania.
